# Specificity in Sociality: Mice and Prairie Voles Exhibit Different Patterns of Peer Affiliation

**DOI:** 10.3389/fnbeh.2018.00050

**Published:** 2018-03-19

**Authors:** Annaliese K. Beery, Jennifer D Christensen, Nicole S. Lee, Katrina L. Blandino

**Affiliations:** ^1^Department of Psychology and Biology, Program in Neuroscience, Smith College, Northampton, MA, United States; ^2^Neuroscience and Behavior Graduate Program, University of Massachusetts, Amherst, MA, United States

**Keywords:** partner preference, social approach, prairie vole, mouse, social behavior, sociability, selective, affiliation

## Abstract

Social behavior is often described as a unified concept, but highly social (group-living) species exhibit distinct social structures and may make different social decisions. Prairie voles (*Microtus ochrogaster*) are socially monogamous rodents that often reside in extended family groups, and exhibit robust preferences for *familiar* social partners (same- and opposite-sex) during extended choice tests, although short-term preferences are not known. Mice (*Mus musculus*) are gregarious and colonial, but in brief laboratory tests of social preference they typically prefer social novelty. This preference for novel vs. familiar peers may represent a species-specific difference in social decision-making between mice and prairie voles. However, the tests used to measure preferences in each species differ markedly in duration and degree of contact, such that the behaviors cannot be directly compared. We assessed whether social preferences for novelty or familiarity differed between mice and prairie voles of both sexes when assessed with matching protocols: the sociability/social preference test (SPT) typically used in mice (short, no direct contact), and the partner preference test (PPT) used in voles (long, direct contact). A subset of voles also underwent a PPT using barriers (long, no direct contact). In the short SPT, behavior did not differ between species. In the longer test, pronounced partner preferences emerged in prairie voles, but mice exhibited no social preferences and rarely huddled. No sex differences were evident in either test. Direct physical contact was required for partner preferences in huddling time in voles, but preference for the partner chamber was evident with or without contact. Both prairie voles and mice are social, but they exhibit important differences in the specificity and extent of their social behavior. While mice are often used to study social approach and other behaviors, voles are a more suitable species for the study of selective social relationships. Consideration of these differences will be important for studies examining the neural mechanisms supporting different kinds of peer social behavior.

## Introduction

Social groups are a common feature of many species; life in such groups can be supported by affiliative interactions among group members, as well as by lack of anti-social behaviors such as aggression and territoriality. Not all social species prefer familiar social contacts and repeated interactions, however. In rodents, selective affiliation between adults is often studied in voles: prairie voles are socially monogamous rodents that show opposite-sex and same-sex preferences for a familiar partner (i.e., partner preferences; Williams et al., 1992; DeVries et al., 1997), and meadow voles live in winter social groups and form enduring, selective partner preferences for adult peers (Beery et al., 2008, 2009; Ondrasek et al., 2015). In contrast, laboratory mice typically prefer novel individuals in brief tests of social interaction (Moy et al., 2004). Because the behavioral tests used in mice and voles differ markedly, it is unknown whether these differences arise from differences in tests or from species-specific differences in social behavior in mice and voles.

Once passed over in favor of larger model organisms, mice are now the most common laboratory mammal—by one estimate accounting for 46% of mammalian research subjects in physiology, up from 4% at the turn of the 20th century and just 6%–10% in the 1980s before the advent of transgenic mouse research (Beery and Zucker, 2011). Despite their popularity as laboratory research models, some social behaviors are not exhibited by mice and therefore cannot be studied in this species. For example, mice are promiscuous breeders, and studies of prairie voles, California mice and other monogamous species have led to many insights about the formation of selective social bonds for mates and how these vary across species (Carter et al., 1995; Donaldson and Young, 2008; Turner et al., 2010; Johnson and Young, 2015). Studying diverse species is also important to determine the variety of pathways supporting behaviors, as well as the generalizability or translatability of findings across species (Donaldson, 2010; Phelps et al., 2010; Taborsky et al., 2015).

Selective partner preferences may be another behavior mice do not display and cannot be used to study. Alternatively, differences in peer-directed social behavior between mice and voles may be an artifact of different testing circumstances. Social preferences in voles are most commonly assessed using the partner preference test (PPT), while social investigation and social interest in mice are most commonly assessed in social interaction with a single novel individual (e.g., File and Seth, 2003), social recognition/habituation tests (e.g., Choleris et al., 2003; Bielsky et al., 2004), or in the three-chambered social approach/preference tests (e.g., Yang et al., 2011).

The PPT was originally developed in the laboratory of Dr. C. Sue Carter (Williams et al., 1992) and assesses the extent of social contact and time in proximity to a partner relative to a stranger. The PPT has been used extensively to assess how different manipulations alter formation and maintenance of *preferences for a mate* in monogamous prairie voles and to a lesser degree in other monogamous species (Ahern et al., 2009; Kingsbury and Goodson, 2014; Carp et al., 2016). The PPT is also used to assess factors affecting social preferences for *same-sex peers* in meadow voles (Beery and Zucker, 2010; Anacker et al., 2016a,b), prairie voles (DeVries et al., 1997), and occasionally other rodents (e.g., Triana-Del Rio et al., 2011). One study has examined long-term social preferences of female mice during an 18 h three-chambered social choice test with stimulus mice housed behind wire mesh (Harrison et al., 2016).

The three-chambered sociability/social preference test (also called the Crawley sociability test) was devised in 2004 as a modification of the PPT and other social tests, specifically oriented toward measuring social approach. It has been widely used to assess both sociability and social preferences in mice (Moy et al., 2004, 2007; Nadler et al., 2004; Schwartzer et al., 2017), with similar tests used in rats (Smith et al., 2015, 2017). To assess sociability, mice are typically given a choice between a novel object (an empty wire pencil cup) and a social stimulus (a pencil cup covering a novel mouse). In order to assess social preference (herein referred to as the social preference test, SPT), mice are presented with one novel and one familiar social stimulus under the pencil cups. In this variant, males and females of multiple mouse strains (including oxytocin null mutants) preferred novel individuals (Moy et al., 2004; Crawley et al., 2007).

There are important differences between the SPT and PPT. Test durations are markedly different at 10 min and 3 h long, respectively. Prior PPT studies have shown that in prairie voles, preferences manifest by the end of the first hour and become significant by the second and third hour, with no enhancement from longer testing intervals (Williams et al., 1992). The PPT also allows for extensive physical contact compared to the SPT. Social stimulus animals in the PPT are tethered around the neck, allowing contact with the focal individual, as well as free movement throughout a portion of the chamber. Thus, social proximity in the PPT refers to huddling time, whereas proximity in the SPT indicates social investigation. Finally, familiar animals in the SPT are typically only briefly familiarized with each other; they are not individuals with which lasting relationships are likely to have formed, reducing the likelihood of detecting preferences based on such relationships. For these reasons, behavior may differ in important ways between these assessments, obscuring our understanding of species-specific differences in behavior.

## Materials and Methods

### Animal Subjects

Prairie voles were bred locally and housed with a same-sex cage-mate from weaning. Voles are photoperiodic and were maintained on a 14:10 h light:dark cycle, consistent with summer conditions. Twelve prairie voles (six male and six female) were used as focal test subjects in the SPT, and 1 week later the PPT.

C57BL/6 and C57BL/10 mice were bred locally and were maintained on a 12:12 light cycle. Mice were weaned into groups of 2–4 and separated to pairs at least 1 week prior to testing. Sixteen mice were used as focal test subjects (eight male and eight female; half of each sex were C57BL/6 and half were C57BL/10).

Additional individuals of matched species, sub-strain and sex were used as social partners or strangers. Tests were conducted at 6.3 ± 0.5 months of age (mean ± SEM). All procedures adhered to recommendations in the Guide for the Care and Use of Laboratory Animals published by the National Research Council, and were approved by the Institutional Animal Care and Use Committee at Smith College.

### Social Preference Tests

**Figure 1:**
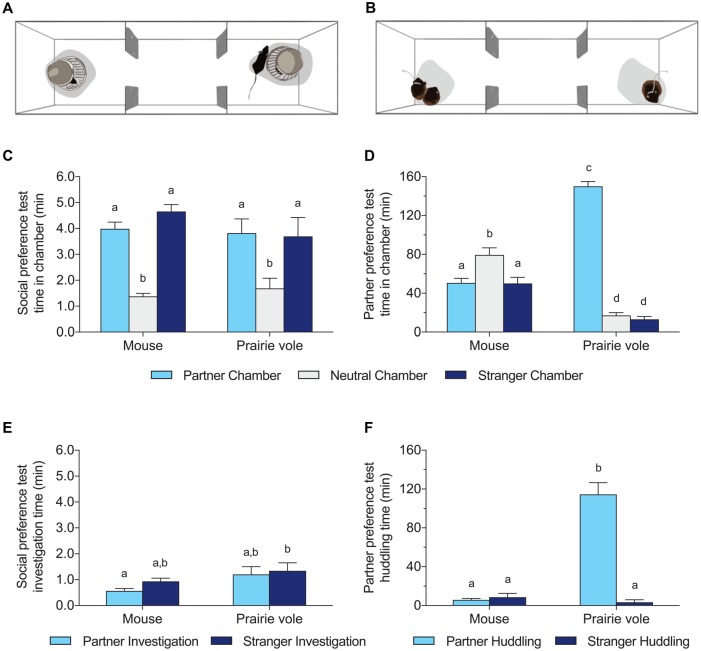
Social behavior differences between mice and prairie voles across test types. **(A)** Schematic version of the 10 min social preference test (SPT), and **(B)** schematic of the 3 h partner preference test (PPT). Both tests were run with both species. **(C,D)** Time spent in the chamber occupied by the familiar partner, no subject, or a stranger in the SPT **(C)** and PPT **(D)**. **(E,F)** Time spent adjacent to the cup (investigation time) or the tethered subject (huddling time) across tests. Different letters above the bars indicate groups significantly different in *post hoc* tests corrected for multiple comparisons.

The social preference version of the three-chambered social approach test was used to assess the inclination to seek social novelty, modeled on Yang et al. (2011). A linear apparatus (20 × 75 × 30 cm) was divided into three equal compartments. Stimulus animals were placed under wire pencil cups (Galaxy pencil holder, Spectrum Diversified) at each end of the apparatus, while the center chamber remained empty (Figure 1A). Two social stimuli were used: a familiar same-sex social partner (the cage-mate) and a novel individual of the same species, sex, and (if relevant) sub-strain as the focal individual and partner. Positions of the familiar and novel stimulus animals were alternated between tests. Familiar individuals were cage-mates of the focal subject and thus even more familiar than in the classic mouse test, as in novelty preference tests in rats (Smith et al., 2015, 2017), and more comparable to the familiar subjects in a PPT. Focal individuals were acclimated to the center of the apparatus for 5 min prior to test onset. Tests lasted 10 min and were video recorded for analysis.

### Partner Preference Tests

PPTs were conducted as described previously (Ahern et al., 2009; Anacker et al., 2016a,b), using the same apparatuses as the SPT. Familiar and novel social stimulus animals were tethered at opposite ends of the apparatus (Figure 1B). Tethered animals were acclimated to the chamber for 5 min before placement of the focal animal in the center neutral chamber. Tethered subjects are typically calm after this duration. Tests lasted 180 min and were video recorded for analysis.

A subset of voles (*n* = 6 males) received a second, modified PPT conducted using pencil cups in place of tethers. These “cup PPTs” were used to distinguish the effects of test duration from the effects of access to full physical contact. Prior research in our lab has shown that voles tested in multiple PPTs exhibit equivalent huddling over time and across tests (Beery et al., 2009).

### Data Analysis

Video recordings were scored using Intervole Timer v1.6 (Annaliese Beery) without knowledge of the partner and stranger positions. Comparisons across species were conducted via two-way analysis of variance (ANOVA) examining effects of species (mouse/vole) and stimulus (partner/stranger) on time adjacent to the stimulus. *Post hoc* tests (Tukey’s HSD) were used to detect differences between all groups. Chamber times were analyzed in the same manner. Although all possible pairs are compared in this method, only 4/6 (adjacent) and 9/12 (chamber) pairings are useful to interpret (e.g., Mouse partner chamber vs. mouse center chamber is useful, but not mouse partner chamber vs. vole center chamber). Partner preference in each group was defined as significantly more time adjacent to the partner than the stranger. Preference score was defined as relative preference for the partner (time adjacent to the partner/time adjacent to the partner+stranger). Preference scores and activity within each test apparatus were compared using *t*-tests across species.

Sexes and strains were used in equal numbers across all conditions and thus pooled. We conducted sub-group analyses to explore effects of sex and strain on behavioral outcomes using *t*-tests between males and females of each species and between C57BL/6 and C57BL/10 mice for each outcome. No statistically significant sex or strain differences were found.

Statistical analyses were performed in JMP 8.0 and GraphPad Prism 7.0. Results were considered significant at* p* < 0.05, and all tests were conducted two-tailed.

## Results

### Species Differences in Social Behavior in the Partner Preference Test

Mice and voles differed profoundly in their behavior in the 3 h PPT (Figures 1B,D,F). Prairie voles spent extensive time in physical contact with their partner (mean 114 ± 12 min), in contrast to mice (mean 5.9 ± 1.4 min). Two-way ANOVA of huddling time showed significant effects of species (mouse/vole), huddling target (partner/stranger), and their interaction (species: *F*_(1,52)_ = 77.28, *p* < 0.0001; huddling target: *F*_(1,52)_ = 84.63, *p* < 0.0001; interaction: *F*_(1,52)_ = 93.59, *p* < 0.0001). Prairie voles exhibited significant preferences for huddling with the partner over the stranger (*p* < 0.0001, Figure 1F), and huddled significantly more with their partners than did mice (*p* < 0.0001, Tukey’s HSD). Mice did not exhibit significant preferences for either the partner or stranger, and spent little time huddling with either subject.

Two-way ANOVA of time spent in each chamber showed significant effects of chamber type (partner/neutral/stranger), and chamber interaction with species (chamber: *F*_(2,78)_ = 83.78, *p* < 0.0001; species: N.S.; interaction: *F*_(2,78)_ = 123.4, *p* < 0.0001). Mice spent significantly more time alone in the neutral chamber than in the partner chamber or the stranger chamber (each *p* < 0.05, Tukey’s HSD). Prairie voles spent significantly more time in the partner chamber than did mice (*p* < 0.0001, Tukey’s HSD), and more time in the chamber with their partner than either the neutral or stranger chamber (each *p* < 0.0001, Tukey’s HSD).

### Partner Preference Expression Requires Physical Contact

**Figure 2:**
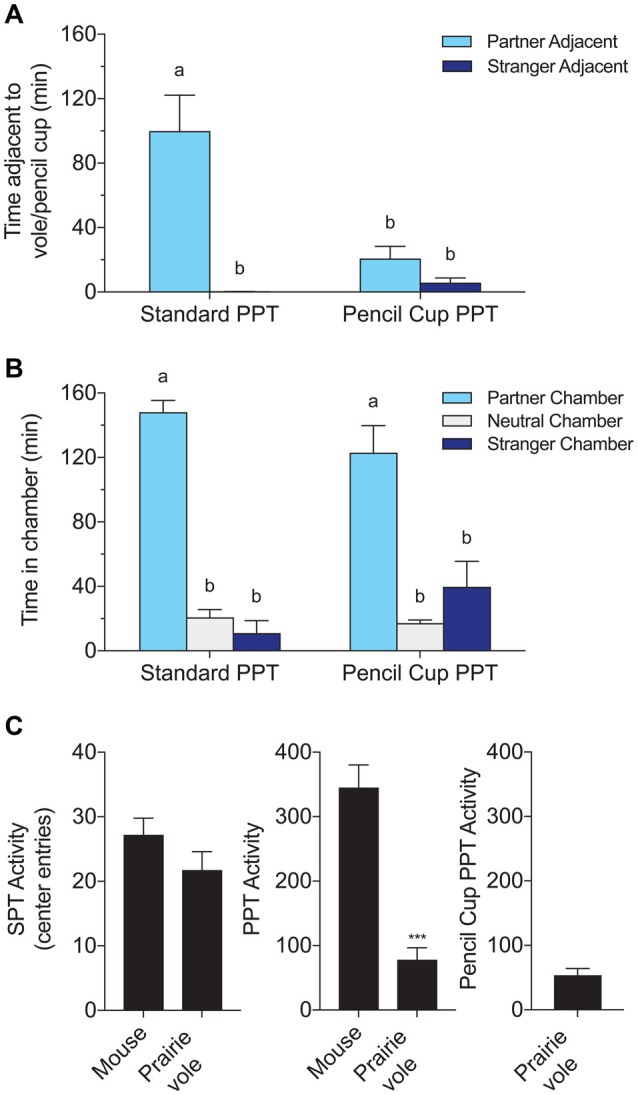
Comparison of a standard PPT involving a tethered partner and stranger to a modified test using wire pencil cups over the stimulus voles. Both versions of the test lasted 3 h. **(A)** Time adjacent to the tethered stimulus subject or pencil cup containing the subject. **(B)** Time in each chamber. Chamber times were equivalent with and without direct contact, but partner preferences in time spent adjacent to the partner were only found in the test that allowed full contact. Letters indicate groups with significantly different means. **(C)** Species comparisons of activity, defined as the number of entries to the center chamber. Mice were significantly more active over the 3-h PPT (***p < 0.0001, *t*-test).

PPTs were compared to modified “cup PPTs” in six male voles to determine whether differences in behavior in the SPT and PPT are due to the difference in duration, difference in physical contact, or both. Access to physical contact strongly affected time in proximity to the partner (Figure 2A). Two-way ANOVA showed a significant effect of test type (PPT/cup PPT), stimulus subject (P/S), and interaction between these factors on time adjacent to a stimulus vole (stimulus subject: *F*_(1,20)_ = 23.47, *p* < 0.0001; test type *F*_(1,20)_ = 9.61, *p* = 0.0056; interaction: *F*_(1,20)_ = 12.82, *p* = 0.002). Partner preferences were only evident in the standard PPT, and partner huddling in the standard PPT was higher than all other outcomes (Figure 2A; multiple comparisons using Tukey’s HSD between all groups, groups with different letters differed significantly).

In contrast, chamber times were equivalent across the two test types (Figure 2B). Two-way ANOVA with chamber (P/N/S) and test type (PPT/cup PPT), and their interaction as factors, yielded a significant effect of chamber (*F*_(2,30)_ = 77.06, *p* < 0.0001) but no effect of test type or interaction between test type and chamber time. *Post hoc* tests revealed that time spent in the partner chamber was significantly higher than other chamber times in both test types, and was indistinguishable across test types (Tukey’s HSD between all groups).

### No Species Differences in Social Behavior in the Social Preference Test

Behavior was more similar between mice and voles in the 10 min SPT with pencil cups. On average, mice spent less time near the partner than the stranger, while voles investigated both subjects roughly equally, but the difference in preference scores was not significant (0.36 ± 0.035 in mice vs. 0.51 ± 0.093 in voles; *t*_(26)_ = 2.06, *p* = 0.12). Focal animals of both species spent more time in some chambers of the SPT than others (Figures 1A,C,E), but there was no effect of species on the distribution of chamber times (two-way ANOVA: effect of chamber *F*_(2,78)_ = 27.20, *p* < 0.0001, no effect of species or interaction). Contact time with the wire cups enclosing stimulus animals differed between species (two-way ANOVA: effect of species *F*_(1,52)_ = 6.560, *p* = 0.013) but there were no significant differences across species in groups matched on stimulus identity (e.g., partner adjacent in mice vs. partner adjacent in voles).

### Activity

Mice were more active/exploratory than voles in the PPT, with 345 ± 35 vs. 78 ± 18 (mean ± SEM) crossings from a side chamber to the center chamber (*t*_(22)_ = −6.75, *p* < 0.0001, Figure 2C). Mice also crossed into the center chamber more often in the shorter SPT, but this difference was not significant (27 ± 3 vs. 22 ± 3 crossings). There was no difference in activity between voles tested in the regular PPT and the same voles tested in the cup PPT.

## Discussion

Mice and voles exhibited pronounced differences in social contact in the PPT, independent of sex and consistent with the existence of strongly selective social preferences for familiar peers in prairie voles (DeVries et al., 1997; Lee et al., unpublished data) but not mice. Voles spent the majority of the test huddling with their partner and over 90% of the test in occupied chambers. Mice did not exhibit significant partner preferences, spent 56% of the test in occupied chambers, and only 8% in social contact with another mouse. In 18-h modified PPTs in wild female mice using wire cage dividers, time in the social chambers was close to 50%, as in the present study, although huddling could not be measured (Harrison et al., 2016). Wild mice also demonstrated more positive behaviors like grooming and sniffing, and less negative social behaviors like fighting and chasing, over the course of 3 days of observed cohabitation (Harrison et al., 2016), and some nursing females form significant associations with other specific females (Weidt et al., 2014). Thus, mice can form relationships in specific circumstances, although they do not exhibit the extensive social huddling and social preference present in voles. Rats also appear to lack preferences for familiar individuals (Schweinfurth et al., 2017). These species-specific differences in social structure indicate the use of different animal models for different social behaviors. In particular, voles are more suitable for the study of selective social relationships.

In the present study, different testing scenarios led to important differences in conclusions about social behavior. Both the duration of the test and the ability to engage in social contact involved in the 3-h PPT shaped the outcomes observed. The major differences between mice and prairie voles in social preference and social contact time detected in long tests of social behavior were not detected in the shorter SPT. Differences between chamber times in the tests lacking physical contact (SPT and cup PPT) in the same voles particularly reinforce the importance of test duration for the expression of partner preferences. While the SPT has proven valuable for assessing sociability and investigation, it does not detect the formation of social relationships. Comparison of the modified “cup” PPTs to standard PPTs demonstrates that the physical contact permitted by the standard PPT was necessary for the expression of partner preferences in time adjacent to the stimulus animals. However, chamber times were similar across the cup and standard PPTs, suggesting that chamber times in this long test can illustrate social preference irrespective of contact. These results are similar to findings in opposite-sex tests of titi monkeys, who showed preferences for proximity to a social partner but not increased contact with an enclosing barrier (Carp et al., 2016).

In our study, the preference for social novelty in mice in the SPT was not significant. Novelty preference may be more pronounced in juvenile mice tested at 6 weeks of age (e.g., Moy et al., 2004), or greater familiarity with the familiar mouse may reduce novelty preference.

As research on social behaviors and disorders grows, mice are increasingly being used to study the hormones, neurotransmitters, and genes involved in different social behaviors, the roles of life experience, and sources of individual variation (e.g., Ferguson et al., 2001; Choleris et al., 2003; Curley et al., 2009; Dölen et al., 2013). As the present study demonstrates, both test format and choice of species impact social outcomes in such studies; long tests in socially selective species such as prairie and meadow voles are important for the study of specific relationships between peers. The use of multiple species with distinct patterns of social behavior should thus provide greater insight into the substrates of peer relationships in diverse mammals including humans.

## Data Availability

The dataset generated for this study is archived at the Open Science Framework website at http://osf.io/j29t5.

## Author Contributions

JDC, NSL and KLB conducted the study and contributed to the design. AKB conceived the study, coordinated the study, analyzed the data, prepared figures and wrote the manuscript. All authors critically revised the manuscript and gave approval for publication.

## Conflict of Interest Statement

The authors declare that the research was conducted in the absence of any commercial or financial relationships that could be construed as a potential conflict of interest.
